# Supplementary material for: Activation of fibroblast growth factor-inducible 14 in the early phase of childhood IgA nephropathy
Source: PLoS One. 2021 Oct 1;16(10):e0258090. doi: 10.1371/journal.pone.0258090 (PMC8486145; doi:10.1371/journal.pone.0258090)
Supplement: Attachment — Submitted filename: Rebuttal letter_2021-6-23.docx [file pone.0258090.s001.docx]

22 June, 2021

Dr. Fabio Sallustio
Academic Editor
PLOS ONE
U.S. Headquarters Public Library of Science
1160 Battery Street, Koshland Building East, Suite 100
San Francisco, CA 94111 United States

Re: Manuscript ID: PONE-D-20-39482

Dear Fabio Sallustio, PhD

Thank you for giving us an opportunity to submit our revised manuscript entitled, “Activation of fibroblast growth factor-inducible 14 in the early phase of childhood IgA nephropathy” (PONE-D-20-39482) to PLOS ONE. We also appreciate the time and effort you and the reviewers have dedicated to provide insightful feedback on ways to strengthen our paper. Thus, it is with great pleasure that we resubmit our article for further consideration. We have incorporated changes that reflect the detailed suggestions you have graciously provided. We also hope that our edits and the responses we provide below satisfactorily address all the issues and concerns you and the reviewers have noted.

To facilitate your review of our revisions, the following is a point-by-point response to the questions and comments delivered in your letter dated, March 29, 2021.

With these changes to the manuscript, we hereby resubmit our revised manuscript for a secondary evaluation.

Thank you once again for your consideration of our paper.

Yours sincerely,

Yuko Tezuka

Corresponding author:

Mariko Eguchi, MD

Department of Pediatrics

Ehime University Graduate School of Medicine

Shitsukawa, Toon, Ehime 791-0295, Japan

Telephone: +81-89-960-5320

Fax: +81-89-960-5941

E-mail: maeguchi@m.ehime-u.ac.jp

**Response to the comments by Academic Editor:**

Thank you for your helpful comments to our manuscript. Our answers to each of your comment are written below.

*[1] The sequencing analysis of human biopsies is not sufficient as reported. A complete transcriptome analysis is needed to show differences between the analyzed sample groups. It would add much value to the study.*

Reply:

We agree with your comment and a usefulness of transcriptome analysis of human biopsied samples in this study. However, because only a small number of preserved FFPE tissue samples of the patients were available for the study, we could not perform transcriptome analysis of human biopsy samples. In the future, however, we would like to proceed with transcriptome analysis for newly diagnosed cases. We have acknowledged this limitation in discussion (Page 29, Lines 447-449; Page 33 Lines 517-520)

*[2] Moreover, also statistical methods and quality control for RNAseq analysis are poorly described and they need to be much more thorough.*

Reply: We have added a description of statistical methods and quality control for RNAseq analysis in materials and methods (Page 19, Lines 263-265; Page 19, Lines 274-278).

*[3] Moreover, as explained in PLOS’s Data Policy, data set must be available at the time of publication. Sequencing Data must be deposited in a public repository and made accessible by everyone by a link or a dataset accession number that must be reported in the methods.*

Reply: We are now taking a step to register our data to DDBJ Sequence Read Archive (DRA) and will be available by public soon. A dataset accession no. will be added in the manuscript as soon as we get the number.

We have removed the phrase including “data not shown” to meet the requirement of your journal.

**Response to the comments by Reviewer #1:**

Thank you very much for your helpful comments. We have answered each of your points below.

<Major points>

*[1] Authors should clarify the different time points in the animal model and the precise number of mice for each group.*

Reply: In this study, three HIGA mice and 2 control mice were sacrificed at 9,11,14,26, or 40 weeks of age to obtain tissue specimens. We used 4 HIGA mice and 3 control mice in the measurement of urinary protein excretion in each week. We have added sentences in the text (Page 14, Line 185; Page 15, Lines 204-205) and Figure2.

*[2] Authors should explain the samples used for any experimental procedures.*

Reply: We have explained the animal models in the materials and methods sections (Page 13, Lines 164-180).

*[3] IgAN patients are classified as Mild and Sever. I suggest reporting MESTC Oxford classification.*

Reply: Thank you for your valuable advice. In this study, we treated patients based on “Guidelines for the Treatment of Childhood IgAN” proposed by the Japanese Society for Pediatric Nephrology. The patients were divided into mild and severe in this guideline. We agree the Oxford classification is more appropriate, therefore, we added MESTC Oxford classification in the text (Page 9, Lines 135-137) and Table 1.

*[4] a crucial point is the potential influence of therapy in the expression of FN14 in human biopsies. In table 1, authors reported all the therapies for each subject. However, it is necessary to specify if patients underwent therapies at the time of kidney biopsies, as this point represents a central issue in the interpretation of results and conclusion.*

Reply: Thank you for your pivotal criticism. No case of steroids or immunosuppressants were used prior to time of renal biopsy. We have added the explanation about the treatment prior to time of biopsy in the paragraph of patient samples (Page 8, Lines 126-127) and discussion (Page 29, Lines 442-444), and Table 1. We have added the details of case 14 (Table 1).

*[5] Moreover, in figure 2 the representation of Fn14 staining in glomerular is unclear. A quantification of the intensity of Fn14 staining is necessary,*

Reply: Thank you for your suggestions. We evaluated the intensity of Fn14 staining in the glomeruli by measuring the proportion of Fn14-stained area in each glomerulus using the ImageJ software and average values were compared at different time points (Page 17, Lines 236-240). The results are shown in Figure 3C (Page 24, Lines 360-365; Page 25, Lines 376-378).

*[6] the quality of images in figure 3 should be improved. IgA staining presents a high background and is too dark. In panel B, all the nuclei of tubular cells seem to be positive for FN14 signal, also in the control. Authors should discuss these results and should clarify the reason why they analyzed Fn14 expression only in glomeruli.*

Reply: We have improved the quality of images in figure 3. We have revised the term (Page 3, Lines 38) and added terms to clarify the sentences (Page 30, Line 466, 468).

According to THE HUMAN PROTEIN ATLAS [https://www.proteinatlas.org/], Fn14 staining by antibody is not detected on cells in glomeruli, but moderate staining is detectable on cells in tubules. Regarding the comment “*all the nuclei of tubular cells seem to be positive for FN14”,* we think these staining is reflecting the physiological staining of Fn14 in the tubules. In pediatric IgA nephropathy, mesangial proliferation in glomeruli is frequently observed findings compared to tubular lesion which is rarely seen because of early stage. Therefore, we analyzed Fn14 expression only in glomeruli in this study.

We added sentences in the paragraph of discussion (Page 32, Lines 505-507).

*[7] The RNAseq results should be validated using Real Time PCR and/or tissue staining*

Reply: Thank you for your suggestion. We tried the validation of RNAseq results by real-time PCR, but sufficient RNA samples for reliable validation with real-time PCR were not available after RNAseq.

*[8] Authors should clarify* *statistic methods for RNAseq results*

Reply: We have described statistic methods for RNAseq results in the paragraph of Materials and Methods (Page 19, Line 274-278)

<Minor point>

*[9] Please, improve the readability of Table 1*

Reply: We have brushed up the items listed in the table to make it readable.

*[10] the whole text needs language revisions*

Reply: We have reviewed and corrected the text with the help of English editing service (Page 34, Line 536-537).

**Response to the comments by Reviewer #2:**

Thank you very much for your comments. Our answers to your points are as follows.

The reference number has been changed when adding some sentences in revised manuscript. We have also added or revised the text to clarify.

[1] *While checking references of this work, in my opinion, the abstract and the introduction and through the manuscript, their statements need to be reformatted by specifically citing what evidence is referenced from animal models and what is cited from human models. On both models, it needs to be specific of what specific kidney cell they are referencing, typical examples are found on lines 74, 75,79,81.*

Reply: We extensively revised and clarified what specific kidney cell referencing on both models in introduction (Page 6, Lines 87-88, 90-91), by describing the specific animal models and human diseases reported (Page 6, Lines 92-93; Page 6-7, Lines 94-98).

<More specific comments>

[2] *Line 58, what % of IgAN patients progress to ESRD and what are the known clinical predictive factors for progression?*

Reply: We have added sentence about the prognosis and clinical and histological predictive factors in the introduction (Page 5, Lines 62-72), mainly about the following points;

Among pediatric patients with IgA nephropathy in Japan, 11% have been reported to develop ESRD within 15 years.

Clinical predictors of a poor outcome were a low GFR, a high mean blood pressure and a high amount of albuminuria at time of biopsy, and low GFR and a high albuminuria during follow up.

Histological predictors were mesangial hypercellularity, endocapillary hypercellularity, tubular atrophy and crescents.

While the incidence of pediatric IgA nephropathy patients who show hypertension or decreased renal function at onset is relatively rare in comparison with adults, proteinuria is the most important risk factor for progression of the disease in childhood, in particular, the degree of proteinuria during the follow-up period.

*[3] Lines 96-98 seem to be repetition of statements on lines 92-94.*

Reply: We have eliminated the sentence corresponds to lines 96-98 (Page 7, Lines 112-114 in revised, markup manuscript).

*[4] Line 113, "high proteinuria" is a non-acceptable term in pediatrics. Acceptable descriptors are nephrotic range (> 1.0 mg/mg in 1st am urine specimen) or non-nephrotic range (0.2 to 1.0 mg/mg).*

Reply: As your suggestion, the term “high proteinuria” is an inappropriate description. In this study, we treated based on “Guidelines for the Treatment of Childhood IgAN” proposed by the Japanese Society for Pediatric Nephrology. In this guideline, amount of urinary protein is stratified by whether early morning urinary protein / creatinine ratio exceeds 1.0 or not, therefore, we have replaced the term “high proteinuria” throughout the paper with “heavy proteinuria” to use more precise term (Page 8, Lines 130). While nephrotic range refers 2.0 or higher of urinary protein / creatinine ratio, we did not use the term “nephrotic range” in our study.

*[5] Line 252, missing commentary addressing type and dose of treatment(s) prior to time of biopsy.*

Reply: We have added explanation about the treatment prior to time of biopsy in the paragraph of patient samples (Page 8, Lines 126-127) and Table 1.

We also have mentioned about the treatment in the discussion (Page, 29, Lines 442-444).

*[6] Lines 292 to 298, difficult to locate on figures.*

Reply: Thank you for your suggestion. We show the ratio of the mean U-pro / Cr in HIGA mice (n = 4) to the mean U-pro/Cr in control mice (n = 3), as 1 at 9 weeks. We have added the annotations of the vertical axis of the graph (Figure 2 B) to “U-pro/Cr, the ratio of HIGA mice to control”. We also have added the text in paragraph of results (Page 22, Lines 317).

Also, since the proliferation of the mesangial matrix was more noticeable than the mesangial cell proliferation, we have revised the sentence to clarify (Page 22, Lines 325-330).

*[7] Line 385. "early stage of IgAN" adds in children. I would suggest to group the limitations of the study. Can't help to wonder why the authors did not do transcriptome analysis of human biopsies. The association of Fn14 deposition early in childhood IgAN biopsies remains loose otherwise. In terms of translational science, it warrants more robust findings and associations. This is purely descriptive, there are no statistical comparisons.*

Reply: Thank you for your important suggestion. We have added “IgAN in childhood” (Page 29, Lines 445). We agree with your comment and consider this point is important. In this study, we used preserved FFPE tissue samples from patients with pediatric IgAN and only small number of samples were available for the experiments. Therefore, we could not perform transcriptome analysis of human biopsy samples. This is the limitation in this study. In the future, however, we would like to proceed with transcriptome analysis for newly diagnosed cases.

We have acknowledged this limitation in discussion (Page 29, Lines 447-449; Page 33, Lines 517-520).

*[8] Table 1. There is no specification on time to biopsy, duration of treatment and time of follow up.*

Reply: We have revised the Table 1 including the period and treatment prior to biopsy, and follow-up period.

Thank you for giving us the opportunity to strengthen our manuscript with your valuable comments and queries.
