# Supplementary material for: Activation of fibroblast growth factor-inducible 14 in the early phase of childhood IgA nephropathy
Source: PLoS One. 2021 Oct 1;16(10):e0258090. doi: 10.1371/journal.pone.0258090 (PMC8486145; doi:10.1371/journal.pone.0258090)
Supplement: Attachment — Submitted filename: Rebuttal_letter 20210907 final.docx [file pone.0258090.s002.docx]

4 September, 2021

Dr. Fabio Sallustio
Academic Editor
PLOS ONE
U.S. Headquarters Public Library of Science
1160 Battery Street, Koshland Building East, Suite 100
San Francisco, CA 94111 United States

Re: Manuscript ID: PONE-D-20-39482R1

Dear Dr. Sallustio,

Thank you for giving us the opportunity to submit our revised manuscript, entitled “Activation of fibroblast growth factor-inducible 14 in the early phase of childhood IgA nephropathy” (PONE-D-20-39482R1), to PLOS ONE. We appreciate the time and effort you and the reviewers have dedicated to provide insightful feedback on ways to strengthen our paper. Thus, it is with great pleasure that we resubmit our article for further consideration. We have incorporated changes that reflect your detailed suggestions. We hope that the revised manuscript satisfactorily addresses all the issues and concerns that were noted.

To facilitate your review of our revisions, the following is a point-by-point response to the questions and comments delivered in your letter dated July 26, 2021.

Thank you once again for your consideration of our paper.

Yours sincerely,

Yuko Tezuka

Corresponding author:

Mariko Eguchi, MD

Department of Pediatrics

Ehime University Graduate School of Medicine

Shitsukawa, Toon, Ehime 791-0295, Japan

Telephone: +81-89-960-5320

Fax: +81-89-960-5941

E-mail: maeguchi@m.ehime-u.ac.jp

**Response to the comments by Academic Editor:**

Thank you for your helpful comment on our manuscript. Our answers to each of your comments are provided below.

*[1] Please, provide in the text of "Methods" the accession Number for your Sequencing Data in the public repository.*

Reply:

1. We have mentioned the accession number for our sequencing data in the public repository in the text of “Materials and methods” (Page 20, lines 282-283).

*[2]* *Please add more details and references about the mouse model of IgAN in the introduction.*

Reply: Thank you for your comment. We have added a description of the mouse model of IgAN in the Introduction (Page 7, lines 109-113) and have also listed the corresponding references (references 30-34). Reference list has also been updated (Page 39, lines 627-639).
